# Transition dynamics modeling of pre-trained accelerometry representations for time to diagnosis of Parkinson’s disease

**DOI:** 10.1007/s10985-026-09721-1

**Published:** 2026-08-03

**Authors:** Huiming Xie, Fei Xue, Xiao Wang

**Affiliations:** Department of Statistics, Purdue University, West Lafayette, Indiana USA

**Keywords:** Parkinson’s disease, Wearable sensors, Self-supervised learning, Markov chain, Survival analysis, 68T07 (Artificial neural networks and deep learning), 62G05 (Nonparametric estimation)

## Abstract

Early identification of Parkinson’s disease is critical for timely intervention. Disruptions in sleep architecture and changes in physical activity patterns have been reported years before motor symptom onset, yet prodromal behavioral changes spanning sleep and daytime activity patterns are subtle and difficult to detect with conventional clinical tools. Wearable sensors provide a scalable means of monitoring these behaviors in natural settings, but extracting meaningful, interpretable features from high-frequency, unlabeled time series remains a major challenge. We present an end-to-end framework for assessing Parkinson’s disease risk from wrist-worn accelerometry via automated feature extraction and survival modeling of time to diagnosis. Behavioral states are derived from unlabeled data using pretrained models including random forests with hidden Markov models for physical activity classification and a self-supervised learning based sleep staging model. We jointly model both sleep stage and physical activity sequences using hierarchical nonstationary Markov chains stratified by time of day and temporal resolution, characterizing individual-level behavioral rhythms and transition dynamics. Gradient-boosted Cox proportional hazards models are then used to estimate Parkinson’s disease risk from these transition-based features. Applied to accelerometer data from the UK Biobank, our approach outperforms baselines that exclude dynamic modeling or rely on traditional functional data analysis, while providing interpretable predictors from long sequences of wearable sensor data. This demonstrates the potential of integrating pretrained AI models, temporal sequence modeling, and survival analysis to detect early behavioral signatures of neurodegeneration.

## Introduction

Parkinson’s disease (PD) is a progressive neurodegenerative disorder that is typically diagnosed only after 50–70% or more of dopaminergic neurons in the substantia nigra have already degenerated (Fearnley and Lees 1991; Zafar and Yaddanapudi 2023; Heng et al. 2023). This substantial neuronal loss, which precedes the onset of hallmark motor symptoms such as bradykinesia, rigidity, and resting tremor (Postuma and Berg 2019), limits the potential for early intervention and highlights the need for sensitive, scalable biomarkers capable of detecting disease at the prodromal stage. A growing body of evidence suggests that multiple non-motor symptoms, such as rapid eye movement (REM) sleep behavior disorder (RBD), constipation, hyposmia, and depression, can emerge years before a clinical diagnosis (Postuma and Berg 2019; Berg et al. 2015). Despite this, efforts to identify reliable prodromal markers have often relied on self reporting, small clinical cohorts and unimodal data sources, limiting their generalizability and clinical utility (Heinzel et al. 2016).

In recent years, digital health technologies have emerged as promising tools to address this gap. Among them, wearable accelerometers have gained traction for their ability to passively and objectively capture physical activity and sleep behavior with high-resolution measurements over the 24-hour cycles in free-living conditions, offering an unobtrusive alternative to traditional clinical assessments (Templeton et al. 2025; Troiano et al. 2008; Matthews et al. 2016). Most notably, wrist-worn accelerometers have been validated against well-established benchmarks for estimating physical activity energy expenditure (Van Hees et al. 2015; Esliger et al. 2011). Their superiority in wear compliance and compatibility with consumer wearables further solidify their role as a robust tool for population-scale behavioral monitoring (Doherty et al. 2017; Shcherbina et al. 2017).

The UK Biobank (UKBB) represents one of the most comprehensive resources for large-scale analysis of wearable sensor data in middle to old aged adults with long-term follow-up and extensive phenotyping and genotyping (Sudlow et al. 2015). Its accelerometry study collected 7-day, 100 Hz triaxial wrist acceleration data from around 100,000 participants (Bycroft et al. 2018). These data are linked to longitudinal diagnostic outcomes, including incident PD, allowing for the first time a rigorous investigation of how subtle, real-world variations in behavior may precede PD diagnosis (Doherty et al. 2017). Importantly, the passive nature of data collection reduces the bias, cost and scalability challenges associated with clinical instruments, and enables monitoring across heterogeneous real-world populations (Bycroft et al. 2018; Doherty et al. 2017).

However, the high-dimensional nature of raw accelerometer data (e.g., 100-Hz triaxial streams over 7 days for the UKBB cohort) makes analysis challenging: downsampling risks obscuring fine-grained physiological signatures, while focusing on high-frequency details may overlook broader circadian context. Preliminary results by Schalkamp et al. (2023) based on the UKBB cohort show that accelerometer data, when analyzed using crafted features from extracted physical activity levels, outperforms data from other modalities including genetic, lifestyle, biochemical and prodromal symptoms data, making it a promising and cost-effective tool for early PD risk identification in the general population and participant selection in neuroprotective therapy trials.

Nevertheless, the potential of these wearable movement-tracking data for identifying early signs of neurodegeneration from the dynamics of physical activities and sleep patterns has yet to be fully explored. Recent efforts in extracting the activity information from accelerometer data include the work of Willetts et al. (2018); Walmsley et al. (2022), who developed a machine learning pipeline combining random forests with hidden Markov models (HMM) to classify physical activities and sleep periods in free-living conditions, trained on the CAPTURE-24 accelerometer dataset (Chan et al. 2024). As for finer-grained sleep stages, Yuan et al. (2024b) introduced a self-supervised learning (SSL) framework for sleep staging, leveraging small polysomnography(PSG)-labeled datasets to train models capable of classifying detailed REM/non-REM sleep stages during sleep time in massive unlabeled accelerometer datasets such as those from the UKBB.

While pretrained models offer promising tools for large-scale, granular activity and sleep annotation, applying these derived labels to identify prodromal PD or uncover biomarkers remains methodologically challenging. First, the relevant behavioral signals, such as sleep-stage transitions or circadian shifts in activity, are subtle and embedded in complex, nonstationary temporal dynamics across multiple timescales. Second, PD onset is rare in population studies and often right-censored, requiring models that can handle sparse, incomplete time-to-event data. Finally, extracting meaningful, generalizable features across these temporal scales while preserving model interpretability crucial for clinical translation remains a key obstacle.

To address these challenges, we develop an end-to-end framework for early PD detection using wrist-worn accelerometry. Our approach comprises three core components. First, we extract interpretable behavioral states from raw data using pretrained models: a self-supervised learning model for sleep staging alongside an activity recognition model for classifying physical activity intensity (e.g., sedentary, light, moderate-to-vigorous activity). Second, we model transitions between these multimodal behavioral states (sleep stages and activity levels) using hierarchical nonstationary Markov chains that capture individual-level dynamics across multiple timescales and circadian phases. Third, we quantify PD risk by incorporating these temporal features into a gradient-boosted Cox proportional hazards model, enabling survival analysis with censored time to PD diagnosis outcomes and interpretable hazard estimates.

Our work makes several main contributions to early detection of neurodegeneration from wearable sensor data. (1) We integrate pretrained sleep staging and activity recognition models into a survival analysis framework, demonstrating how these techniques such as self-supervised learning enhance time-to-event prediction while preserving interpretability via hazard-based feature representations. (2) We propose a novel Markov-survival framework that quantifies how disrupted circadian behavioral rhythms, including sleep-stage transitions and activity fluctuations, influence long-term neurodegeneration risk. (3) On a large-scale UK Biobank cohort, our method outperforms conventional survival models and functional data approaches by jointly leveraging sleep and activity dynamics, under censoring and class imbalance. (4) Critically, our approach operates on raw accelerometer data without manual feature engineering, mitigating information loss from preprocessing and enabling scalable early detection in real-world settings.

Together, these contributions demonstrate how combining pretrained AI models, temporal transition modeling, and survival analysis enables biologically grounded and clinically meaningful insights from raw wearable sensor data. Our approach opens new avenues for population-level screening of prodromal PD and bridges the gap between passive digital sensing and interpretable risk stratification in real-world settings.

## Foundations of the analysis

This section describes the data sources and behavioral representations used in our analysis. Raw accelerometer recordings from the UK Biobank provide continuous measurements of human movement over extended periods, offering a unique opportunity to study long-term behavioral patterns in large populations. However, the high-frequency nature of these signals makes direct statistical modeling challenging. To obtain interpretable representations suitable for downstream analysis, we first transform the raw accelerometry signals into sequences of behavioral states describing both daytime activity and sleep dynamics. These derived behavioral sequences form the inputs to the temporal modeling framework introduced in Section 3.

### Data source and study population

We analyzed data from the UK Biobank (UKBB), a large prospective population cohort study that enrolled more than 500,000 individuals aged 40–69 years between 2006 and 2010 across 22 assessment centers in the United Kingdom (Sudlow et al. 2015; Allen et al. 2012). The study collects extensive phenotypic and genotypic data, incorporating questionnaire responses, physical measurements, biological assays, imaging data, genetic information, and long-term clinical follow-up through linkage with national health records.

#### Accelerometer data collection

Between 2013 and 2015, a randomly selected subset of UKBB participants were invited to participate in a 7-day wrist-worn accelerometry study. Participants wore an Axivity AX3 triaxial accelerometer on their dominant wrist while maintaining their usual daily routines. The device recorded raw acceleration signals at 100 Hz with a dynamic range of *± 8g*. Devices were mailed to participants and configured to begin recording at 10 a.m. two working days after postal dispatch.

Approximately 106,000 participants returned devices with usable recordings. After standard quality-control procedures, including device calibration checks and minimum wear-time requirements (at least 72 hours of valid data), approximately 96,600 participant recordings were retained for analysis (Doherty et al. 2017).

**Fig. 1:**
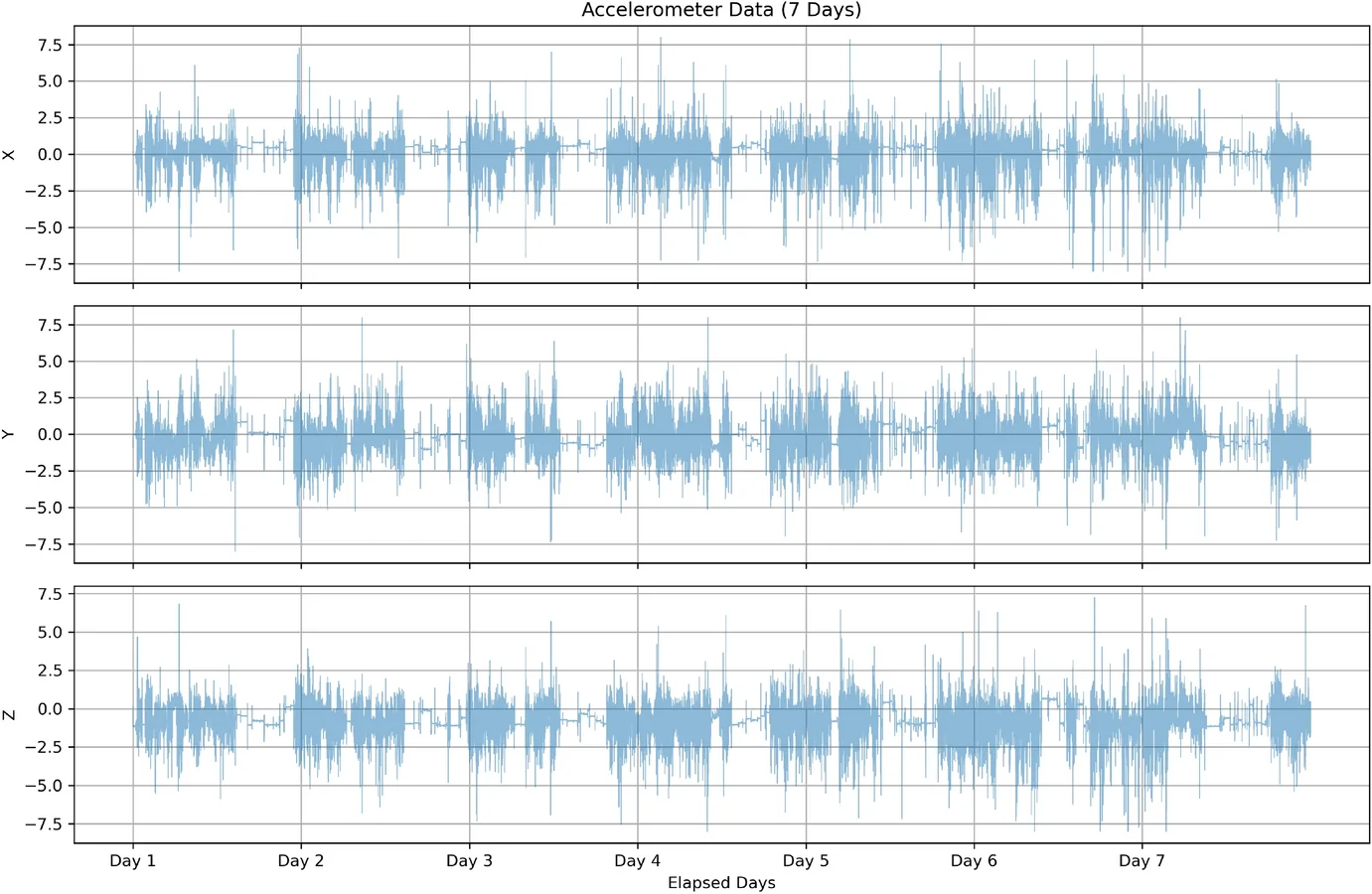
Seven-day raw triaxial accelerometer recording from a randomly selected UK Biobank participant

Figure 1 illustrates the raw triaxial accelerometer signals for a randomly selected participant over a 7-day period. The three orthogonal acceleration axes exhibit clear diurnal periodicity associated with daily sleep–wake cycles and activity patterns. While these high-frequency signals contain rich behavioral information, their scale and dimensionality present substantial challenges for direct statistical modeling. Common strategies involve a trade-off: downsampling facilitates learning overall trends but can discard informative high-frequency signals, while focusing solely on the raw high-frequency data tends to capture microscale dynamics at the expense of macroscale context. A methodology integrating both perspectives holds considerable promise. Additionally, achieving interpretability alongside the extraction of complex patterns remains difficult. Motivated by these challenges, we propose a systematic feature extraction framework that is domain-agnostic, avoids handcrafted features, and offers generalizability across diverse applications.

#### Data for prodromal Parkinson’s disease

Clinical outcomes in the UKBB are continuously updated through linkage with multiple health data sources including hospital episode statistics, primary care records, death registries, and participant self-reports. Parkinson’s disease (PD) diagnoses were identified from these linked health records. These diagnoses have not been clinically validated and have incomplete coverage, but it has been observed that PD prevalence and incidence in the UKBB matched expected population estimates (Schalkamp et al. 2023).

As of February 2024, approximately 0.96% of the overall UKBB cohort had a recorded PD diagnosis. Within the accelerometry subsample, 211 participants were diagnosed with PD at the time or within two years of the first day of their accelerometer measurement (mean years since diagnosis to acceleration measurement *2.38 ± 3.98* years), and 383 participants were diagnosed more than two years after data collection, representing the prodromal PD group (mean time to diagnosis *5.41 ± 1.80* years). The remaining participants were treated as controls and censored at the last available follow-up date (February 2024). Our study focuses on modeling the time to PD diagnosis in this prodromal group, including the large undiagnosed cohort as controls (censored in February 2024 in the data we used).

**Fig. 2:**
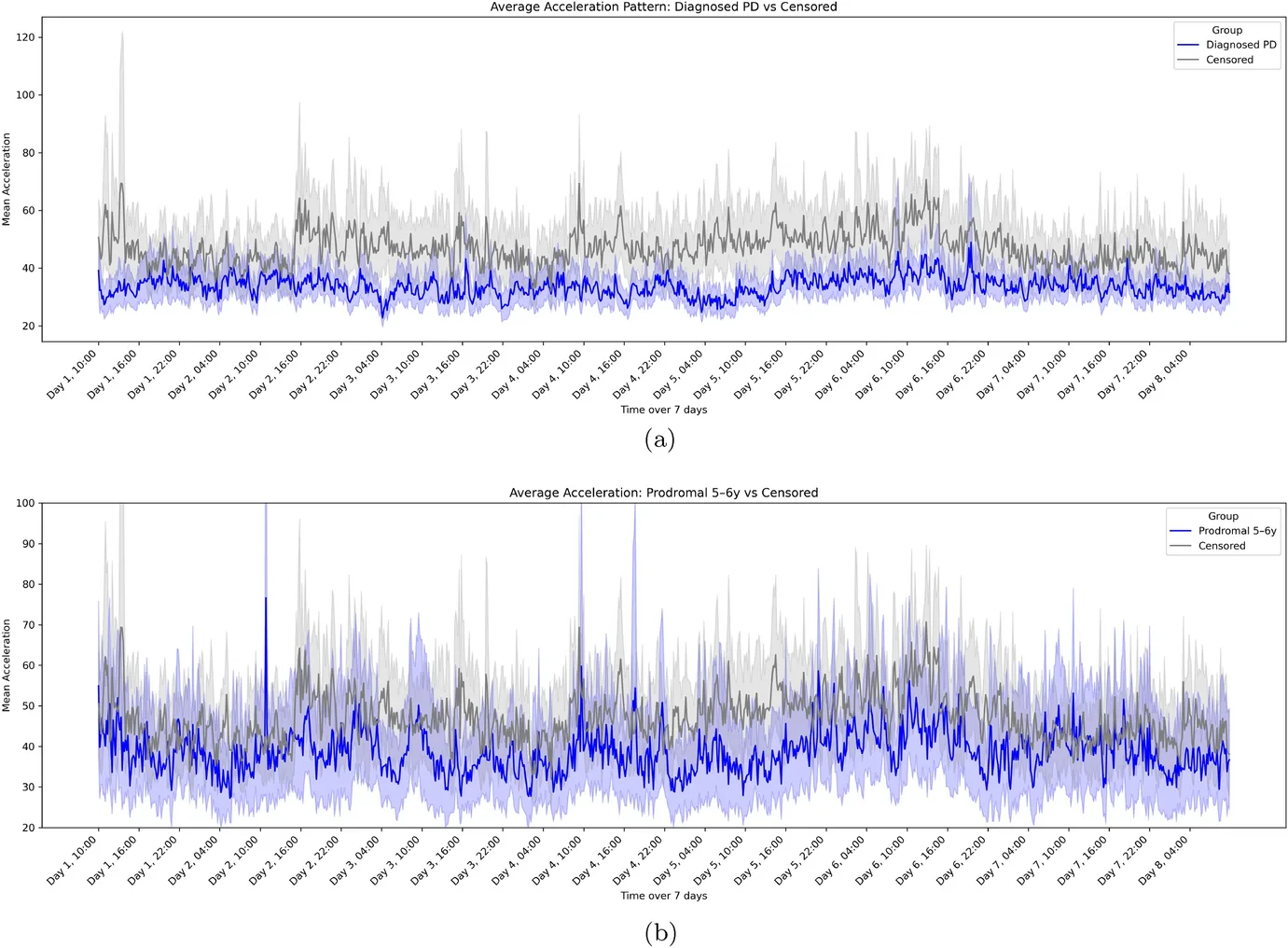
Average acceleration (95% confidence intervals) by diagnostic group: **a** diagnosed PD vs. censored; **b** prodromal 5–6 year vs. censored

To provide an initial descriptive comparison across groups, we examined average acceleration magnitude across PD diagnostic categories, including diagnosed cases, prodromal subgroups stratified by time to diagnosis, and matched control groups. Individuals with diagnosed PD tended to exhibit lower overall activity levels compared with controls (Figure 2a). However, differences between prodromal groups and controls were considerably smaller (see Figure 2b for the prodromal 5–6 year group as an example; plots for other prodromal groups are provided in Appendix C). These patterns suggest that simple summary measures of activity may not adequately capture subtle behavioral changes preceding PD diagnosis and highlight the need for better prodromal differentiation. We address this challenge by developing interpretable, data-driven methods to better assess the risks in the prodromal phase, thereby enabling earlier intervention.

### Behavioral annotation with pre-trained models

To transform raw accelerometry signals into interpretable behavioral representations, we leveraged two pretrained models that infer behavioral states from wrist-worn accelerometer data. These models capture complementary aspects of daily physiology by characterizing both daytime physical activity and nocturnal sleep structure.

Daytime activity states were obtained using the UK Biobank accelerometer analysis pipeline, which applies a supervised machine-learning classifier with temporal smoothing to assign activity labels to short windows of accelerometer data (Doherty et al. 2017; Walmsley et al. 2022). This approach produces 30-second resolution labels for four activity categories: sleep, sedentary behavior, light activity, and moderate-to-vigorous physical activity (MVPA).

To characterize sleep architecture in greater detail, we additionally applied a pretrained sleep-stage classification model based on a self-supervised learning (SSL) framework developed by Yuan et al. (2024b). This model learns general-purpose representations from large-scale unlabeled accelerometer data and is subsequently fine-tuned using polysomnography-labeled datasets to predict sleep stages including wake, rapid-eye-movement (REM) sleep, and three stages of non-REM sleep.

Together, these models produce labeled behavioral sequences that describe both waking activity patterns and sleep stages across the 7-day monitoring period. These behavioral state sequences provide a unified representation of daily behavioral rhythms and serve as the primary inputs to our downstream modeling framework, which aims to characterize temporal behavioral dynamics associated with prodromal PD risk.

Additional details regarding the accelerometer preprocessing procedures and pretrained behavioral annotation models are provided in Appendix A and Appendix B.

## Method

Our proposed approach leverages pretrained and externally validated models to annotate raw triaxial accelerometer data with behavioral states, including activity intensities and different sleep stages, enabling granular characterization of movement patterns linked to prodromal PD. These pretrained annotation models are existing methods and are used here solely as preprocessing tools to convert raw accelerometer signals into behavioral state sequences. Without reliance on domain-specific or handcrafted features, our methodological contribution begins with a transition dynamics framework that extracts interpretable features directly from sequences of activity and sleep-stage labels to capture temporal patterns such as shifts between states in different time periods of the day and using different time scales. We then train an XGBoost-Cox proportional hazards model on the derived features to predict time to PD diagnosis in the general population cohort.

### Modeling dynamic sleep stage transitions using hierarchical nonstationary Markov chains

To characterize the complex, nonstationary temporal structure of human behavior over the 24-hour cycle using the labels predicted from the pre-trained models, we propose a hierarchical nonstationary Markov chain framework. This model accounts for circadian modulation via time-of-day stratification, behavioral variability across timescales through multi-resolution transition dynamics, and distinct state spaces for sleep and activity, preserving physiological interpretability. This structure allows us to flexibly capture both micro-level transitions (e.g., sleep stage shifts, brief activity bouts) and macro-level behavioral patterns (e.g., consolidated sleep, daytime activity) for understanding prodromal signs of neurodegeneration.

#### The state space

We model physical activity levels and sleep stages using two parallel, yet independently estimated, Markov chains. The physical activity state space is defined as:

$$\mathcal {S}_\text {activity} = \{\text {Sedentary (S)}, \text {Light (L)}, \text {Moderate-to-Vigorous (M)}\}.$$

These states reflect movement intensity during wake periods and, derived from the classification models pre-trained on labeled wearable sensor data as introduced in Section 2.2.

The sleep state space is defined as:

$$\mathcal {S}_\text {sleep} = \{\text {Wake (W)}, \text {N1}, \text {N2}, \text {N3}, \text {REM}\}.$$

This taxonomy aligns with standard polysomnographic sleep staging (Berry et al. 2012). N1, N2, N3 represent progressively deeper stages of non-REM (NREM) sleep, distinguished by increasing synchronization in electroencephalography (EEG) activity and reductions in arousability. REM sleep, in contrast, is characterized by vivid dreaming, rapid eye movements, and muscle atonia. These sleep-stage labels for each 30-second epoch in the accelerometer data can be obtained by applying the pretrained SleepNet based on SSL (described in Section 2.2 and Appendix B) to the raw accelerometer data. As in other time periods, some sleep segments contain non-wear intervals longer than 60 minutes. These segments lack meaningful signal for inferring sleep states and cannot be straightforwardly imputed using the average of comparable time-of-day observations, as is commonly done for daytime physical activity levels. We therefore retain the non-wear category as an additional state in the sleep state space.

#### Time-of-day stratification

Having defined the behavioral state spaces for activity and sleep, we now model how transitions between these states evolve across the 24-hour cycle. As human behavior follows robust circadian rhythms governed by the suprachiasmatic nucleus and influenced by environmental cues such as light exposure and social scheduling (Duffy and Czeisler 2009; Mistlberger and Skene 2004), to capture this temporal structure, we stratify the 24-hour day into four non-overlapping intervals that reflect major circadian phases:

$$\mathcal {T} = {\left\{ \begin{array}{ll} \text {Night} & [00{:}00, 06{:}00) \\ \text {Morning} & [06{:}00, 12{:}00) \\ \text {Afternoon} & [12{:}00, 18{:}00) \\ \text {Evening} & [18{:}00, 24{:}00) \end{array}\right. }.$$

For each time segment $\tau \in \mathcal {T}$, we calculate the total time of each state defined in state spaces in Section 3.1.1, and estimate a separate transition matrix during each time segment to allow for time-varying dynamics. For instance, sleep transitions dominate the night interval, while physical activities are more prevalent during other periods. By stratifying transitions temporally, we explicitly accommodate circadian nonstationarity in behavioral state evolution.

This stratification also facilitates clinical interpretability, as deviations from typical circadian transition patterns (e.g., increased REM fragmentation at night, reduced MVPA in the afternoon) may signal early-stage neurological changes relevant for modeling of time to PD diagnosis.

#### Multi-timescale transition modeling

While time-of-day stratification captures how behavioral transition patterns vary across circadian phases, it does not address the fact that these patterns also unfold at different temporal resolutions. Human behavior exhibits structure across a hierarchy of timescales, from second-to-second shifts to hour-long bouts, which can carry distinct physiological and clinical relevance. To jointly capture circadian variation and temporal granularity, we define transition matrices at two complementary resolutions as follows.

##### Fine-scale (30-second) transitions

These transitions represent rapid, moment-to-moment behavioral fluctuations and are particularly suited to capturing transient states such as brief arousals from sleep or spontaneous physical movement.

Let $c \in \{\text {act}, \text {sleep}\}$ denote the state-space category, where $\mathcal {S}_\text {activity} = \{s^{\text {activity}}_1, \dots , s^{\text {activity}}_{K_\text {activity}}\}$ is the set of activity states, and $\mathcal {S}_\text {sleep} = \{s^{\text {sleep}}_1, \dots , s^{\text {sleep}}_{K_\text {sleep}}\}$ is the set of sleep states including a non-wear state, as specified in Section 3.1.1, with $K_\text {activity} = 3$ and $K_\text {sleep} = 6$. Let $S^{\text {fine},c}_t \in \mathcal {S}_c$ denote the fine-scale state process of category *c* at time *t*, and let *i,j* index the states in $\mathcal {S}_c$. The time-of-day bin is denoted by *τ* from Section 3.1.2. We define the fine-scale, time-of-day–specific transition probability matrix for category *c* as

$$\textbf{P}^{(\tau ,\text {fine},c)}_{ij} = \mathbb {P}\!\left( S^{\text {fine},c}_{t+30\textrm{s}} = s^{c}_j \,\bigg |\, S^{\text {fine},c}_t = s^{c}_i,\, t \in \tau \right) .$$

##### Coarse-scale (30-minute) transitions

These transitions encode broader behavioral rhythms and longer-range temporal dependencies, reflecting consolidated sleep bouts or sustained activity. We derive the coarse-scale states by majority voting over 60 fine-scale epochs within each 30-minute block. This smoothing operation mitigates noise and emphasizes sustained behavioral patterns:

$$\textbf{P}^{(\tau ,\text {coarse},c)}_{ij} = \mathbb {P}\!\left( S^{\text {coarse},c}_{t+30\textrm{m}} = s^{c}_j \,\bigg |\, S^{\text {coarse},c}_t = s^{c}_i,\, t \in \tau \right) ,$$

with notations defined analogously as for fine-scale transitions. The dual-resolution modeling enables the system to learn rich features that would be missed by single-scale approaches, such as both micro-arousals and prolonged disruptions in sleep continuity.

To estimate the transition probabilities, we made use of frequency counts of each pairwise transitions, that is, for each participant, and for each combination of time-of-day *τ* and scale $k \in \{\text {fine}, \text {coarse}\}$, the estimated empirical transition probabilities are:

$$\begin{aligned} \hat{\textbf{P}}^{(\tau ,k,c)}_{ij} = \frac{N^{(\tau ,k,c)}_{ij}}{\sum _{j'} N^{(\tau ,k,c)}_{ij'}}, \end{aligned}$$

where $N^{(\tau ,k,c)}_{ij}$ denotes the number of observed transitions from state $s_i$ to $s_j$ in time window *τ* at scale *k* for state-space category *c*.

### Survival analysis with XGBoost-cox for time to PD diagnosis

The multi-timescale, time-of-day–stratified transition matrices from the previous section, together with the total time spent in each activity category and sleep stage, denoted $N_i^{(\tau ,k,c)}$ for state $s_i$ in time window *τ* at scale *k* for state-space category *c*, form a structured representation of each participant’s behavioral dynamics. To assess the prognostic value of these dynamics for PD onset, we use them as inputs to a survival analysis framework.

Given the potential complexity and nonlinearity of the relationship between behavioral patterns and neurodegenerative disease risk, and the rarity of PD diagnoses in population-based cohorts, we adopt XGBoost with a Cox proportional hazards objective as our primary modeling approach. This method combines the flexibility of gradient-boosted decision trees with the ability to model censored time-to-event data using the Cox partial likelihood, enabling it to capture nonlinear relationships and high-order interactions (Chen and Guestrin 2016; Li et al. 2022). Compared to traditional linear Cox models, XGBoost-Cox can accommodate more complex, non-linear feature–response mappings without imposing restrictive assumptions. It also offers improved generalization and regularization relative to deep learning–based models such as DeepSurv, which typically require extensive tuning and a larger number of events (PD diagnoses in this case) to avoid overfitting (Katzman et al. 2018). Similarly, survival random forests tend to suffer from higher variance in low-event settings, as their reliance on fully grown trees can lead to instability when splitting on sparse event data (Ishwaran et al. 2008; Wang and Li 2017). XGBoost-Cox offered a good balance of modeling flexibility, robustness to rare events, and empirical performance in this context (Mayr and Schmid 2014).

We frame the problem as a survival analysis task with the following definitions. For each participant *i*, let $b_i$ denote the baseline accelerometer measurement date, $d_i$ the PD diagnosis date (if observed), and *e* the administrative censoring date. The survival time $T_i$ represents time-to-diagnosis:

$$T_i = {\left\{ \begin{array}{ll} d_i - b_i & \text {if diagnosed} \\ \infty & \text {otherwise.} \end{array}\right. }$$

Participants without a diagnosis by *e* are censored at $C_i = e - b_i$. The observed outcomes consist of $(t_i, \delta _i)$, where $t_i = \min (T_i, C_i)$ is the observed time and $\delta _i = \mathbb {I}(T_i \le C_i)$ the event indicator. We model the hazard function:

$$\begin{aligned} \lambda (t|\textbf{x}_i) = \lambda _0(t)\exp \left( f(\textbf{x}_i)\right) {,} \end{aligned}$$

where $\lambda _0(t)$ is the baseline hazard and *f(· )* is an ensemble of regression trees learned by optimizing the regularized Cox partial likelihood:

$$\begin{aligned} \mathcal {L}(\theta ) = -\sum _{i:\delta _i=1}\left( f(\textbf{x}_i) - \log \sum _{j\in R(t_i)} \exp (f(\textbf{x}_j))\right) + \gamma T + \alpha \Vert \omega \Vert _1 + \frac{1}{2}\lambda \Vert \omega \Vert ^2{.} \end{aligned}$$

here, $R(t_i) = \{j | t_j \ge t_i\}$ represents the risk set at time $t_i$, *T* the number of tree leaves, and *ω* the leaf weights. In the general XGBoost framework, tree complexity can be controlled through regularization terms involving the number of tree leaves and the magnitude of leaf weights. In our implementation, the corresponding regularization parameters were fixed at their default values (*γ = 0*, *α = 0*, *λ = 1*), so no explicit penalty on the number of tree leaves was imposed. Instead, tree complexity was controlled through structural hyperparameters such as the maximum tree depth and minimum child weight, which constrain the growth of individual trees. The leaf weights were estimated during training via gradient-based optimization and were not treated as hyperparameters. Hyperparameter tuning was conducted via grid search within each training fold of a 5-fold stratified cross-validation procedure. The parameter space was defined as follows: learning rate $\eta \in \{0.01, 0.03, 0.05\}$, maximum tree depth $d \in \{2,3,4\}$, minimum child weight $w_{\min } \in \{5,10,15\}$, subsample ratio $\rho \in \{0.4, 0.6, 0.8\}$, and column subsample ratio $\psi \in \{0.4, 0.6, 0.8\}$. The optimal hyperparameters were selected by maximizing the concordance index:

$$\begin{aligned} \text {C-index} = \frac{\sum _{i<j} \mathbb {I}(t_i< t_j)\mathbb {I}(\hat{f}(\textbf{x}_i)> \hat{f}(\textbf{x}_j))\delta _i}{\sum _{i<j} \mathbb {I}(t_i < t_j)\delta _i}. \end{aligned}$$

The final selected values for the hyperparameters were: learning rate *η = 0.01*, maximum tree depth *d = 3*, minimum child weight $w_{\min } = 10$, subsample ratio *ρ = 0.8*, and column subsample ratio *ψ = 0.8*.

We note that although we focus on the timing of clinical diagnosis rather than the latent onset of PD, this approach still serves as a valuable tool for stratifying risk. In practice, the estimated time to diagnosis can highlight individuals whose behavioral patterns signal elevated concern, enabling earlier clinical attention even if the exact onset remains unobservable.

## Real data analysis

To evaluate the proposed method on UKBB accelerometer data, we extracted 30-second and 30-minute transition pairs alongside marginal label counts for sleep stages and physical activity states within each 6-hour time window. For each participant, we computed normalized label counts and estimated transition probabilities (as defined in Section 3.1.3), generating a total of 360 input features for the XGBoost-Cox model.

To benchmark our approach, we compared it against several baselines that use time-series information through downsampled hourly averages of normalized acceleration magnitude. These baselines included: (1) using raw hourly time series as input features for XGBoost-Cox, (2) applying functional principal component analysis (FPCA) for dimensionality reduction with a Cox model, and (3) smoothing the data via B-spline basis expansions before fitting a Cox model.

To further investigate the contributions of different feature types, we conducted ablation studies by progressively removing the estimated transition probabilities, referred to as “transitional features", from our original features, then removing the remaining coarse-grained temporal features, i.e., the counts of each 30-minute state at this point, also referred to as “hierarchical features", and finally removing the features encoding circadian structure by aggregating features across all time periods for each state, which can be considered as removing “periodical features".

Additionally, we assessed model robustness under different covariate settings by conducting parallel experiments either including age and gender as covariates using the subset of participants with available covariates or excluding covariates entirely using all participants with valid data for activity and sleep data prediction. The covariates were incorporated into the XGBoost-Cox model through simple feature concatenation with the behavioral dynamic features.

Using 5-fold cross-validation stratified by diagnosis events, we evaluated our hierarchical Markov transition model against the ablated models and baseline approaches. Model performance was measured using three metrics: (1) the concordance index (C-index) for overall discriminative ability in ranking participants’ risk of disease onset, (2) time-dependent AUC (area under the ROC curve), evaluated from 2.5 to 7 years, following the practice of Schalkamp et al. (2023), which reflects how well the model distinguishes between individuals who experience the event by a certain time point and those who do not, accounting for censoring, and (3) AUPRC (area under the precision-recall curve) computed across all observed events without time stratification, which is informative for imbalanced datasets by emphasizing correct identification of the minority class (events) rather than overall ranking or time-specific discrimination.

### Performance results and comparisons

**Table 1:** Performance comparison with baseline models: mean (standard deviation) on 5-fold cross validation

Method	C-index	Mean AUC	PRAUC
Proposed	**0.817(0.032)**	**0.855(0.042)**	**0.054(0.025)**
+ covariates	**0.827(0.007)**	**0.857(0.028)**	**0.051(0.024)**
XGBoost-Cox	0.723(0.034)	0.760(0.024)	0.019(0.006)
+ covariates	0.776(0.023)	0.787(0.003)	0.018(0.004)
FPCA	0.727(0.018)	0.760(0.022)	0.018(0.004)
+ covariates	0.782(0.015)	0.797(0.023)	0.017(0.002)
B-spline	0.728(0.019)	0.774(0.011)	0.019(0.004)
+ covariates	0.790(0.013)	0.801(0.014)	0.019(0.003)

Bold values indicate the best performance for each metric

The method “XGBoost-Cox" denotes using XGBoost-Cox for the downsampled hourly averages of the normalized acceleration, “FPCA" denotes the method using FPCA on the averages followed by a Cox proportional hazard model, and similarly for “B-spline". “+ covariates” indicates results with covariates. The covariates include age and gender

*Comparison with Baseline Methods* Based on the C-index and mean AUC (averaged time-dependent AUC) and PRAUC metrics (Table 1), our proposed method demonstrated consistent advantages over conventional approaches. Traditional methods using hourly-averaged acceleration time series showed significantly lower performance across all metrics. Specifically, our model achieved superior time-dependent AUC compared to functional data analysis approaches (FPCA and B-spline expansions) applied to the same hourly data as well as XGBoost-Cox applied to the hourly data. For baseline methods, we also experimented with averaging acceleration data every 10 minutes instead of every hour, but increasing the temporal resolution of baseline methods to 10-minute averages reduced performance (for instance, time-dependent AUC went down to around 0.6), likely due to the fact that finer-grained data captured more short-term noise while obscuring the broader circadian patterns most relevant for detecting neurodegenerative changes. Alternative survival approaches (random survival forests and DeepSurv) failed to converge effectively with our proposed features, consistent with the theoretical limitations in low-event settings discussed in Section 3.2.

**Table 2:** Performance comparison with ablated models (with features progressively removed, denoted by “-”: mean (standard deviation) on 5-fold cross validation

Method	C-index	Mean AUC	PRAUC
Full Model (Proposed)	**0.817(0.032)**	**0.855(0.042)**	**0.054(0.025)**
+ covariates	**0.827(0.007)**	**0.857(0.028)**	**0.051(0.024)**
– Trans.	0.783(0.029)	0.840(0.031)	0.040(0.017)
+ covariates	0.811(0.010)	0.848(0.023)	0.044(0.025)
– Trans./Hier.	0.780(0.023)	0.834(0.030)	0.050(0.028)
+ covariates	0.807(0.010)	0.845(0.028)	0.040(0.026)
– Trans./Hier./Per.	0.770(0.022)	0.820(0.037)	0.045(0.026)
+ covariates	0.811(0.013)	0.850(0.006)	0.041(0.019)

Bold values indicate the best performance for each metric

The covariates include age and gender. “+ covariates” indicates results with covariates. Here, “Trans.", “Hier.", and “Per." denote transitional features, hierarchical features, and periodical features, respectively

*Comparison with Ablated Models* The ablation study (Table 2) revealed a clear hierarchy of component importance in our model. The full configuration achieved a mean C-index of 0.82, with exclusion of transition dynamics causing the most substantial performance drop (from 0.82 to 0.78 without covariates), underscoring their critical role in capturing neurodegenerative signatures. Hierarchical structure removal resulted in a moderate AUC reduction of 0.03, while eliminating periodical stratification yielded further declines without covariates. This performance gradient suggests that micro-scale behavioral transitions encode the most discriminative signals for early detection, with hierarchical and temporal features providing complementary refinement.

**Fig. 3:**
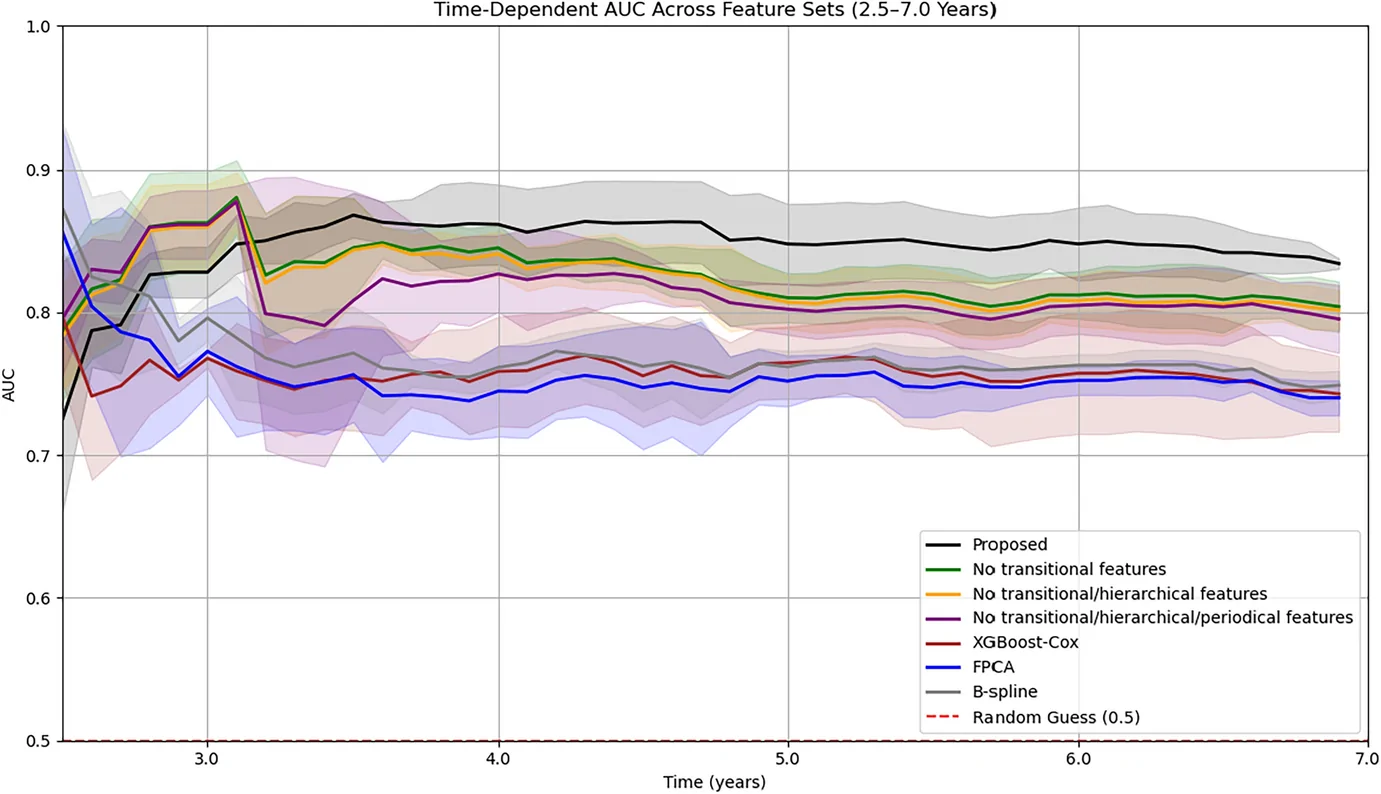
Plot of time-dependent AUC for different models without using covariates (average values ± standard deviations across 5 folds on test sets). The comparison includes all ablated models and baseline models corresponding to Table 2 and Table 1.

**Fig. 4:**
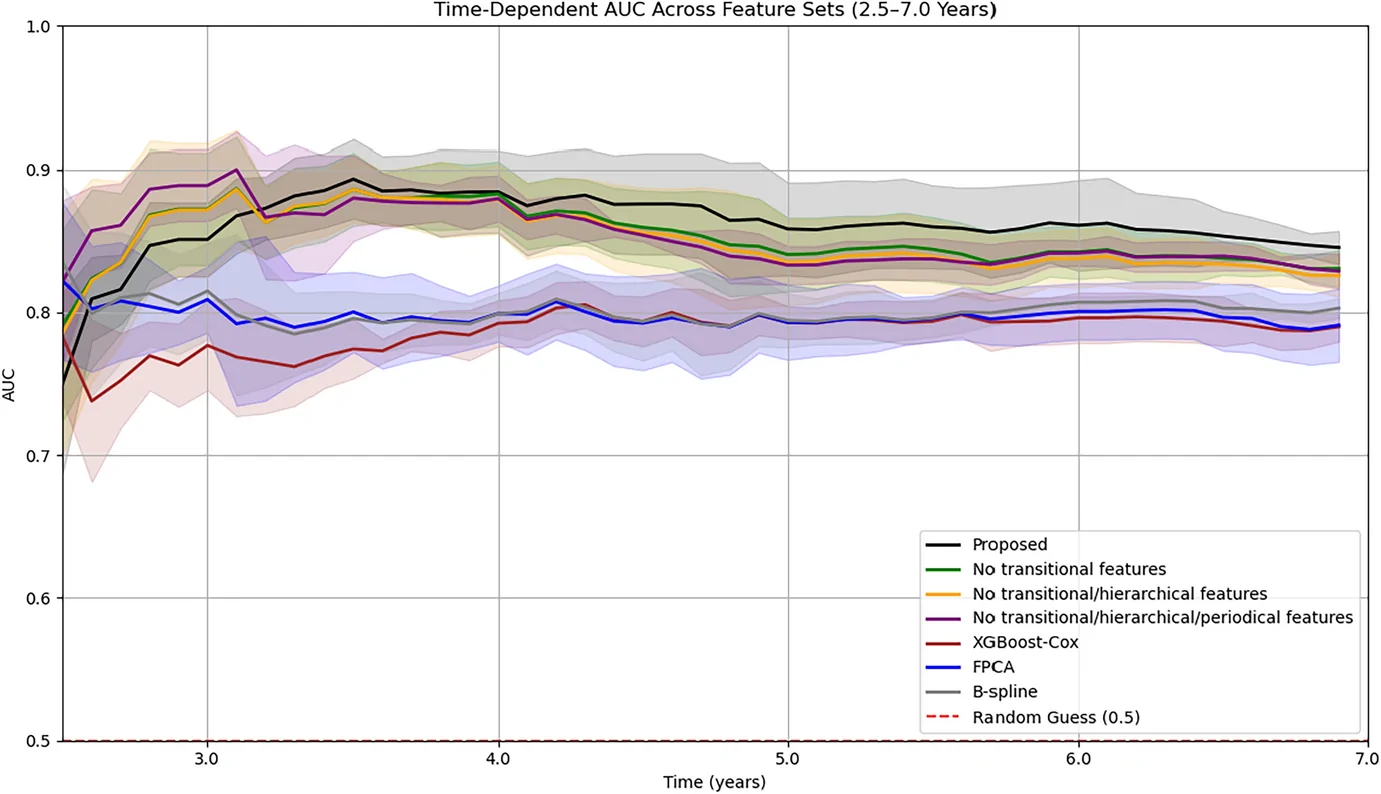
Plot of time-dependent AUC for different models using gender and age as covariates (average values ± standard deviations across 5 folds on test sets). The comparison includes all ablated models and baseline models corresponding to Table 2 and Table 1.

*Temporal Performance Analysis* The time-dependent AUC trajectories (Figure. 3 and 4) exhibit a clinically significant temporal pattern. While our method demonstrates comparable performance to alternatives in the early prediction window (2.5–3 years), its predictive advantage becomes increasingly pronounced in later time windows. This progression suggests a fundamental shift in the underlying predictive features: early performance appears driven primarily by current behavioral states, whereas longer-term predictions benefit substantially from transition dynamics that capture progressive neurodegenerative changes. Notably, the delayed emergence of sleep architecture features as discriminative predictors (after 3 years) closely mirrors established PD pathophysiology timelines. This temporal performance profile parallels observations from lifestyle-based predictors (Schalkamp et al. 2023), where features such as alcohol and smoking status, daytime sleepiness, adiposity and cardiovascular measures, and socioeconomic indicators similarly show stronger predictive value in later stages. The superior late-stage performance carries important implications for PD screening protocols. The method’s strong 7 year AUC suggests particular utility for mid-term risk stratification, filling a critical gap between immediate clinical assessment and long-term population studies.

Notably, while incorporating age and gender covariates improved performance substantially for baseline methods, our approach showed only marginal gains, yet still outperformed all ablated variants. This demonstrates that the transition dynamics inherently capture (though are not limited to) predictive signals that typically require explicit demographic adjustment. More importantly, the results suggest our method simultaneously encodes both demographic-associated and independent pathological signatures, highlighting the unique predictive power of behavioral transition patterns beyond conventional covariates.

### Feature contributions to model predictions

#### Feature importance from XGBoost ranking metrics

We assessed feature importance in XGBoost using five built-in metrics: (1) *Weight*, the frequency with which a feature is used for splits across all decision trees, (2) *Gain*, the average improvement in model accuracy (loss reduction) from splits using the feature, (3) *Cover*, the average number of samples affected by those splits, (4) *Total Gain*, the cumulative accuracy gains from all splits involving the feature, and (5) *Total Cover*, the total number of samples impacted across the model. While Gain and Total Gain emphasize a feature’s predictive power, Cover and Total Cover reflect its breadth of influence across the dataset. For this ranking, we re-trained the proposed model on the full dataset without reserving a hold-out test set, and report the top ten features by each metric in Table 3 and Table 4.

Across metrics, the (30-second) N2 *→* Wake transition during the night consistently emerged among the highest-ranked features. This finding aligns with established sleep physiology: N2 sleep, a light non-REM stage, is particularly susceptible to brief arousals and awakenings, especially in age-related sleep fragmentation (Berry et al. 2012). The prominence of this transition is supported by its physiological plausibility (dominance in later sleep cycles when sleep is lighter), its empirical frequency in hypnogram data, especially in older adults and during microarousals, and prior evidence linking sleep fragmentation to early neurodegenerative processes (Hunt et al. 2022; Minakawa 2022). Its predictive power for time to PD diagnosis may therefore reflect prodromal instability in sleep regulation.

Other frequently identified sleep-related predictors include 30-minute N2 *→* N3 or N2 *→* NA transition at night, (30-second) N2 *→* Wake transition at night. The N2 *→* N3 transition, particularly at the 30-minute resolution, represents healthy deepening into slow-wave sleep, which is associated with metabolic clearance, memory consolidation, and neuroprotective functions (Mander et al. 2017). In contrast, transitions from deeper stages (e.g., N3 *→* Wake) indicate sleep disruption or fragmentation, which has been linked to elevated risks of neurodegenerative and cardiometabolic diseases (Lim et al. 2013; Bonnet and Arand 2003). Night-time non-wear (NA) periods, when accurately detected, may correspond to subtle awakenings or brief device removal, and can be interpreted similarly to Wake to some extent in terms of sleep integrity. Transitions such as (30-second) Wake *→* N3 in the morning or NA *→* N2 in the evening may reflect compensatory sleep or circadian phase shifts, both of which can signal atypical or disrupted sleep patterns.

Notably, physical activity features typically rank highly when aggregated over time (e.g., total duration of a state during a daily period), whereas sleep-related predictors more often appear as state-transition features rather than cumulative times in a given stage. This distinction underscores the value of modeling sleep as a dynamic process, where the sequence and timing of transitions carry predictive information for prodromal PD risk. The inclusion of both fine-grained (30-second) and coarse-grained (30-minute) transition features is empirically justified, as both scales contributed prominently to survival model performance: the fine scale captures rapid state switches and microarousals, while the coarse scale summarizes broader patterns of sleep structure and fragmentation.

**Table 3:** Top 10 features by XGBoost importance (Split-Level Metrics)

Weight	Gain	Cover
night_N2_to_W	30min_night_REM	night_REM
evening_L	evening_S_to_L	30min_night_REM
night_N3_to_NA	30min_night_N2_to_N3	night_N3_to_W
30min_night_N2_to_NA	night_N2_to_W	afternoon_L_to_S
30min_night_N2_to_N3	evening_L	afternoon_S_to_L
morning_S	30min_morning_W	morning_NA_to_N3
afternoon_L_to_S	morning_W_to_N3	afternoon_S
afternoon_S	afternoon_S	evening_N3_to_N1
night_W_to_NA	night_N3_to_W	evening_S_to_L
night_W_to_N3	morning_S	morning_L

**Table 4:** Top 10 features by XGBoost importance (Cumulative Metrics)

Total gain	Total cover
night_N2_to_W	night_N2_to_W
30min_night_N2_to_N3	evening_L
evening_L	30min_night_N2_to_NA
30min_night_N2_to_NA	30min_night_N2_to_N3
morning_S	night_N3_to_W
morning_W_to_N3	morning_S
afternoon_S	afternoon_L_to_S
night_W_to_N3	afternoon_S
night_N3_to_NA	evening_NA_to_N2
night_W_to_N3	morning_W_to_N3

**Fig. 5:**
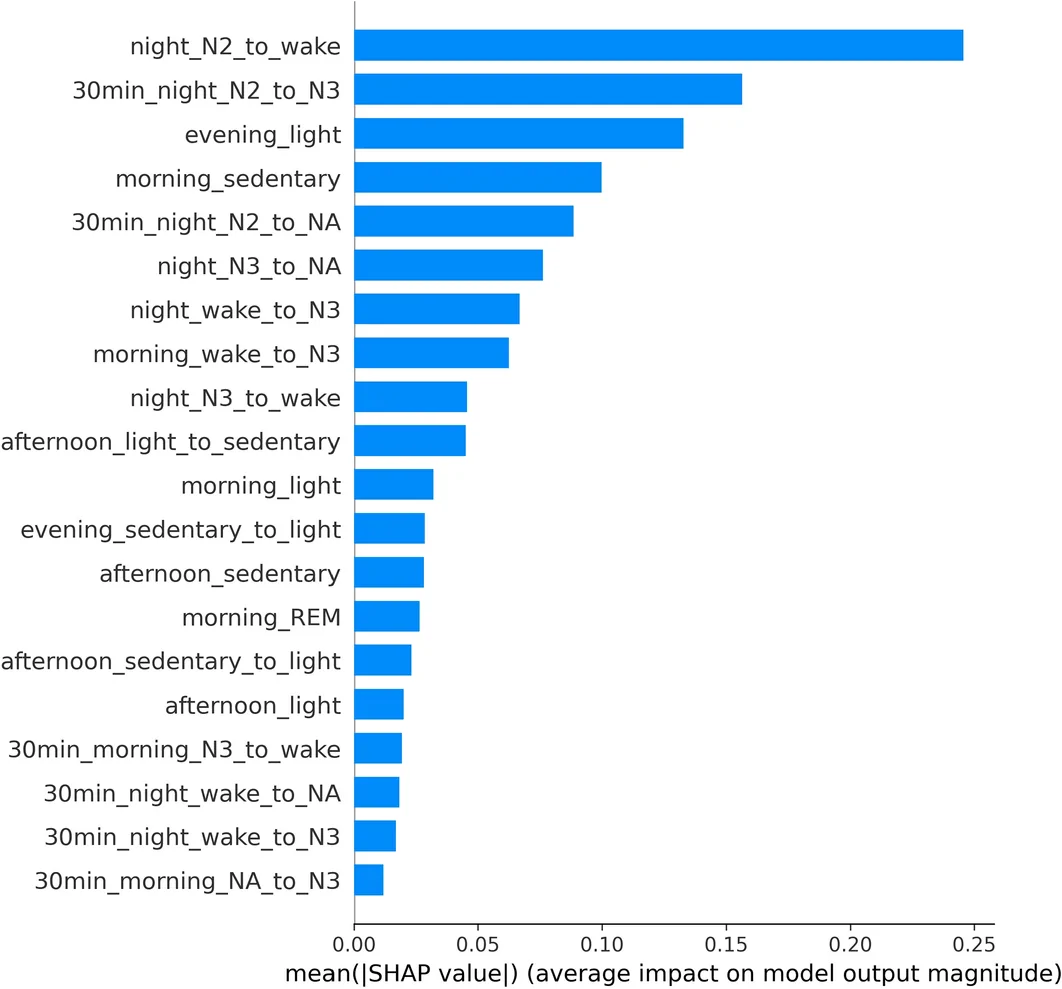
Mean absolute SHAP values for the top 20 predictive features. Each bar represents the average magnitude of the feature’s contribution to the predicted hazard (risk of PD diagnosis), aggregated across all individuals. Features with larger SHAP values are more influential in the survival model

**Fig. 6:**
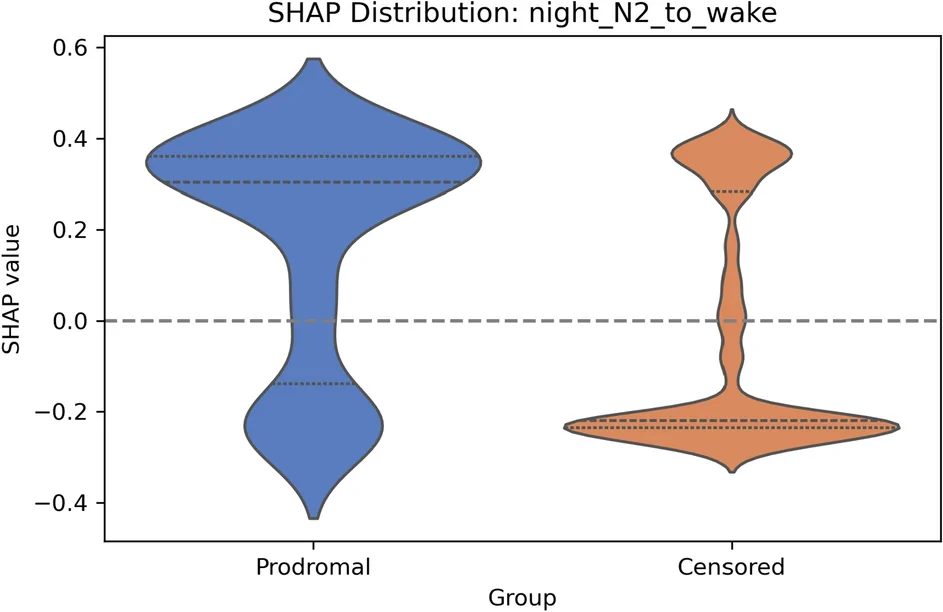
Distribution of SHAP values for the feature night_N2_to_wake stratified by group (Prodromal vs. Censored)

#### SHAP values for directional effect and magnitude

We also used SHAP values (SHapley Additive exPlanations) to quantify how individual behavioral features contribute to the predicted risk of prodromal PD diagnosis. SHAP values decompose a model’s prediction into additive contributions from each feature, using Shapley values from cooperative game theory. This approach is particularly useful for interpreting complex, nonlinear models such as gradient-boosted survival trees, where traditional parametric coefficients are unavailable.

The SHAP summary bar plot (Figure 5) ranks features by their mean absolute SHAP values, which represent the average contribution magnitude across individuals. Larger mean absolute SHAP values indicate features that consistently exert greater influence on model predictions. The top features closely match those identified by XGBoost’s built-in importance metrics (Table 3), with night-time (30-second) N2 *→* Wake and 30-minute N2 *→* N3 transitions among the most important features.

To explore directional effects, we examined the SHAP value distribution for the highest-ranked feature, night_N2_to_wake (night-time 30-second transition of N2 *→* Wake), stratified by outcome in Figure 6. In individuals later diagnosed with PD, this feature frequently had large positive SHAP values, indicating an increased predicted risk; in healthy individuals, SHAP values were typically negative, indicating a protective association. This divergence does not imply separate group-specific coefficients. Because tree-based models like XGBoost capture nonlinear dependencies and conditional interactions, the effect of night_N2_to_wake depends on its interplay with other factors such as overall sleep architecture, activity fragmentation, or circadian timing. The group-level separation in SHAP distributions likely reflects interaction effects, whereby night_N2_to_wake elevates risk only in multivariate behavioral contexts more common in prodromal individuals.

Because transition-based features map directly onto interpretable physiological events, transition matrices offer natural targets for monitoring and intervention. Detecting influential transitions such as N2 *→* Wake, alongside related patterns, could serve as measurable endpoints in clinical trials. This interpretability advantage over raw acceleration-based approaches supports their direct translation to preventive neurology practice.

## Discussion

Our study introduces a hierarchical nonstationary Markov framework to model multimodal behavioral transitions (sleep stages and physical activity levels) from wrist-worn accelerometry, demonstrating through UK Biobank’s large-scale wearable sensor data cohort that dynamic sleep and activity patterns have the potential of improving Parkinson’s disease risk prediction over conventional approaches. By leveraging pretrained machine learning models for activity recognition and pretrained self-supervised learning models for sleep staging, our method automates feature extraction without manual feature engineering, preserving interpretability while overcoming a key challenge in wearable sensor data analysis. Critically, the framework captures macro-scale circadian rhythms and micro-scale disruptions in both sleep architecture and activity fluctuations, enabling clinically meaningful insights without sacrificing predictive power. While sleep transitions dominate risk prediction, integrating activity dynamics refines stratification, particularly for individuals with subtle or heterogeneous prodromal presentations. This balance between utility and interpretability addresses a critical gap in digital biomarkers, positioning our framework as a bridge between raw sensor data and clinical decision-making.

While our model predicts time to PD diagnosis rather than true disease onset, this remains clinically meaningful for risk stratification. In the analysis of UK Biobank wearable sensor data, our model’s performance peaks at a 3–7 year prediction horizon, aligning with PD’s prodromal phase. This temporal pattern suggests two key implications. (1) The method is particularly suited for identifying individuals at elevated risk within a clinically actionable window, enabling targeted monitoring or early intervention. (2) The gradual divergence in sleep and activity transitions, such as worsening sleep stability and daytime activity fragmentation, mirrors PD’s prodromal progression, where subtle dysregulation precedes overt symptoms. Activity transitions, though secondary to sleep, may provide complementary signals for individuals with less pronounced sleep disturbances.

Several limitations merit consideration. While the 30-second epochs from the pretrained models strike a balance between interpretability and temporal detail, ultra-short events (e.g., brief arousals or activity bursts) may require finer temporal resolution to be captured accurately. Pretrained models for activity and sleep stage classifications also have potential for improvement in performance as the methods advance. In addition, these pretrained models may need recalibration for diverse populations beyond the UK Biobank’s demographics, which currently consist predominantly of a Caucasian cohort. Observed transitions may reflect both pathology and adaptive behaviors, complicating mechanistic inference. While first-order Markov assumptions prioritize interpretability, future work could incorporate higher-order dependencies or latent states to potentially capture more complex sleep architecture. Future work could also integrate multimodal sensors to include factors such as heart rate variability or test the framework in prospective intervention studies to validate its real-world clinical impact.

In conclusion, by combining pretrained AI models, temporal transition analysis, and machine learning-enhanced survival modeling, our work advances scalable, interpretable prodromal Parkinson’s disease risk stratification from wearable sensor data. The method’s emphasis on behavioral rhythms rather than isolated symptoms aligns with the systemic nature of the disease, offering a template for studying other neurodegenerative disorders. With the growing adoption of wearable sensors, such frameworks will be vital for transforming passive sensing into clinically actionable tools.

## Figures and Tables

**Fig. 7:**
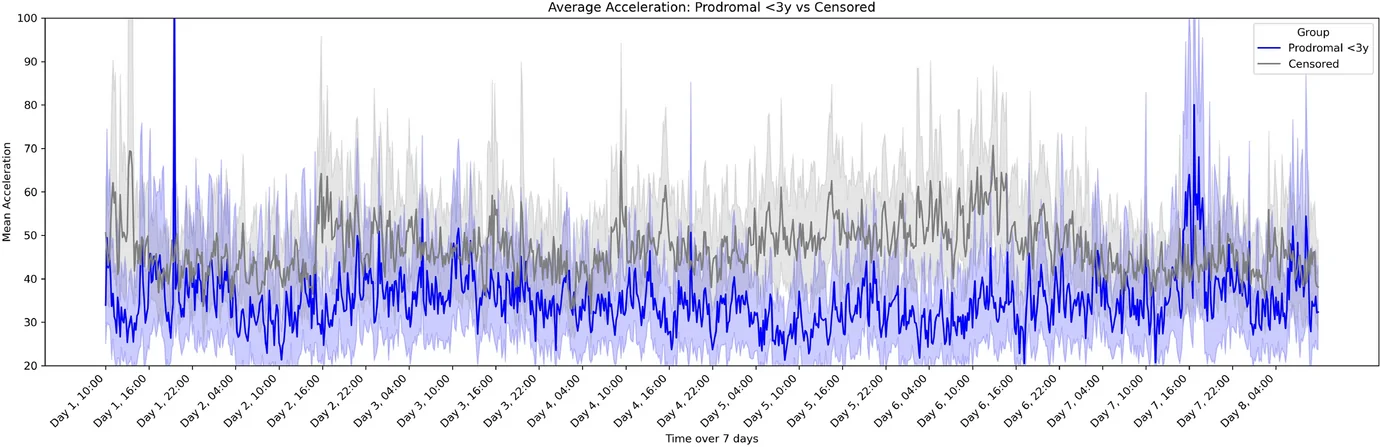
Average acceleration (with 95% confidence intervals) by diagnostic group: prodromal <3 year vs. censored

**Fig. 8:**
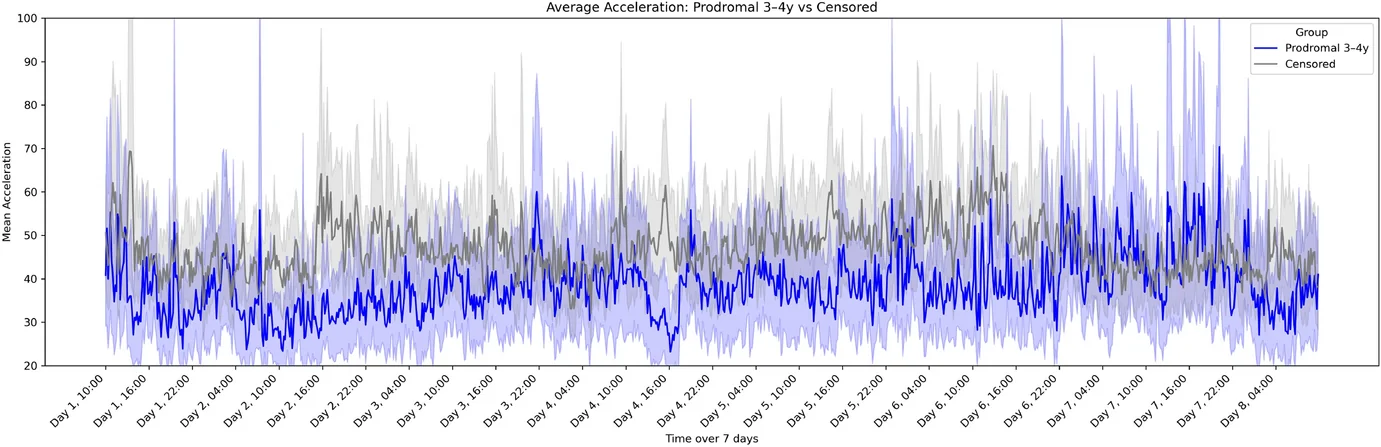
Average acceleration (with 95% confidence intervals) by diagnostic group: prodromal 3-4 year vs. censored

**Fig. 9:**
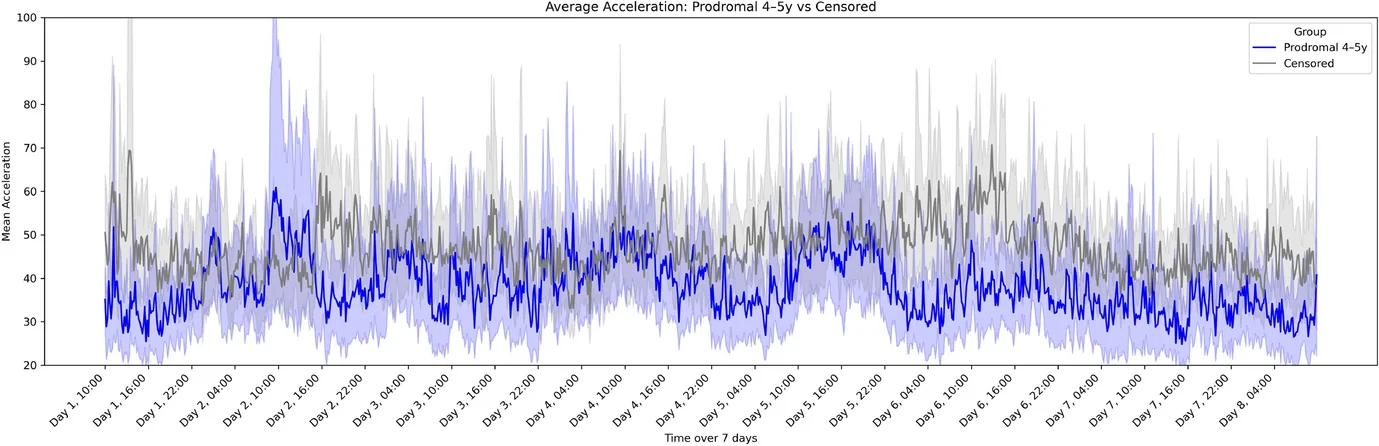
Average acceleration (with 95% confidence intervals) by diagnostic group: prodromal 4-5 year vs. censored

**Fig. 10:**
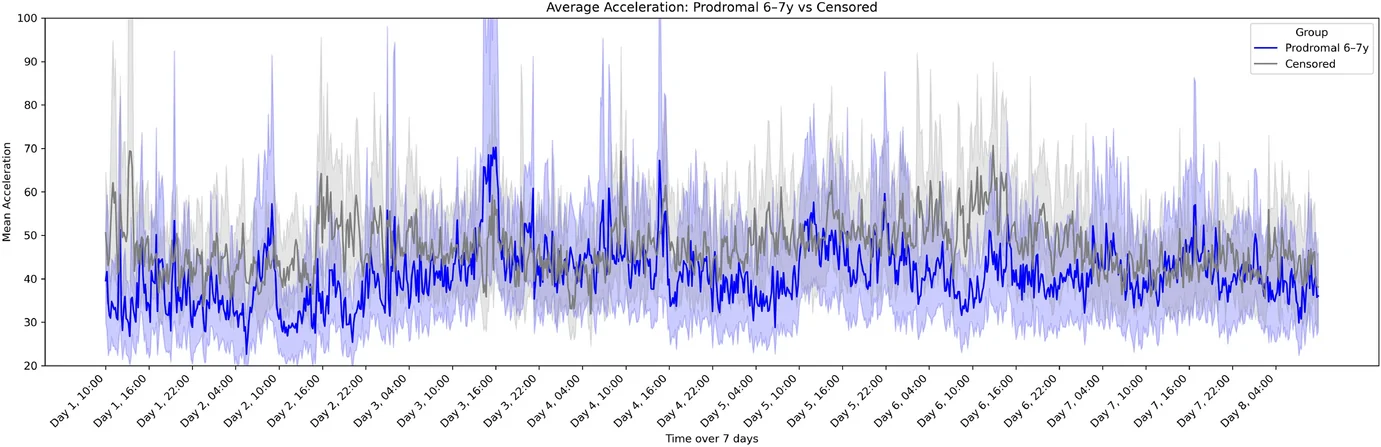
Average acceleration (with 95% confidence intervals) by diagnostic group: prodromal 6-7 year vs. censored

**Fig. 11:**
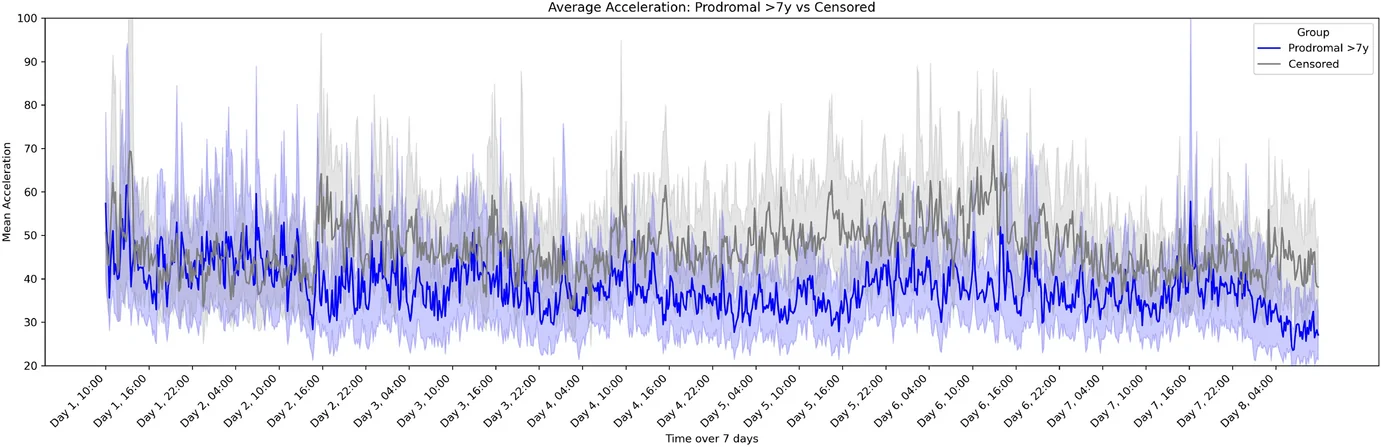
Average acceleration (with 95% confidence intervals) by diagnostic group: prodromal >7 year vs. censored

## A. Accelerometer preprocessing and physical activity classification

Raw accelerometer signals from the UK Biobank were preprocessed following the established pipeline described in Doherty et al. (2017). Raw triaxial acceleration signals were first calibrated to local gravity to correct for device bias and orientation differences.

The 30-second level physical activity labels–*sleep*, *sedentary*, *light* and *moderate-to-vigorous physical activity* (MVPA) are derived from raw wrist-worn accelerometer data by adopting a statistical machine learning pipeline using the UK Biobank Accelerometer Analysis Toolkit (Doherty et al. 2017; Willetts et al. 2018; Walmsley et al. 2022). This approach combines a supervised classification model with temporal smoothing via a hidden Markov model (HMM), trained on free-living accelerometer data from the CAPTURE-24 study (Chan et al. 2024), which provides synchronized camera-derived ground truth activity labels. Unlike traditional approaches based on Metabolic Equivalent of Task (MET)-derived acceleration thresholds, which can be sensitive to device placement and produce noisy transitions, this method yields more accurate and temporally coherent activity labels. The method has been validated in free-living movement behaviors with mean classification accuracy 88% (Walmsley et al. 2022).

In this approach, the classification stage employs a balanced random forest model to assign activity labels to 30-second windows of the triaxial acceleration data. Input features include time- and frequency-domain summary statistics such as vector magnitude, signal entropy and dominant frequency. Formally, given a sequence of input feature vectors $\{\textbf{x}_1, \ldots , \textbf{x}_L\}$, where *L* denotes the number of 30-second windows in the recording, the random forest classifier produces marginal activity predictions $\{\hat{y}_1, \ldots , \hat{y}_L\}$, where $\hat{y}_l \in \mathcal {S} = \{\text {sleep}, \text {sedentary}, \text {light}, \text {MVPA}\}$. To enforce temporal consistency, a first-order HMM is applied, where the latent states $\{z_l\}_{l=1}^L$ correspond to the true (smoothed) activity classes. The model defines transition probabilities $P(z_l \mid z_{l-1})$ and emission probabilities $P(\hat{y}_l \mid z_l)$ derived from the classifier outputs. The most probable sequence of latent activity states is inferred using the Viterbi algorithm (Forney 2005):

$$\hat{\textbf{z}} = \arg \max _{\textbf{z}} P(z_1) \prod _{l=2}^L P(z_l \mid z_{l-1}) \prod _{l=1}^L P(\hat{y}_l \mid z_l).$$

This helps to yield a temporally smoothed sequence of activity labels. In practice, despite instructions for continuous wear over the 7-day period, some periods of non-compliance inevitably occur. Stationary periods (*x*/*y*/*z* *sd < 13mg)* with a duration greater than 60 min are considered as non-wear. Non-wear periods were imputed using the mean value of time-matched data points from other days in the recording, calculated separately for each behavioral prediction. We used these acquired activity labels in subsequent modeling.

## B. Self-supervised learning framework for sleep stage classification

Sleep-stage labels were generated using the self-supervised learning (SSL) framework proposed by Yuan et al. Yuan et al. (2024b). This approach uses a multi-task paradigm to learn generic and versatile representations directly from raw accelerometer data (Haresamudram et al. 2022).

The self-supervised learning framework for sleep stage classification can be viewed as a two-step process. The first step involves self-supervised learning through a designed multi-task classification framework, trained on massive unlabeled raw accelerometer data from UKBB. The raw tri-axial accelerometry was first resampled into 30 Hz (higher than the presumed 20 Hz Nyquist rate for most human activities with frequency less than 10 Hz) and the accelerometry sequence was then divided into consecutive 30-second windows. Let $X(t) \in \mathbb {R}^{3 \times T}$ denote a triaxial accelerometer window spanning *T* samples at 30 Hz (*T=900* for 30-second epochs). An encoder $f_\theta : \mathbb {R}^{3 \times T} \rightarrow \mathbb {R}^d$ maps input windows to a *d*-dimensional latent space (*d=1024*, for dimension of the learned feature vector) using a 1D adaptation of the ResNet-V2 architecture with 18 layers with 10*M* parameters (He et al. 2016).

To train the encoder effectively within this multi-task framework, the model was tasked with solving three complementary temporal pretext classification problems (Yuan et al. 2024a; Saeed et al. 2019). Each task involves identifying whether a particular temporal transformation has been applied to the input window, encouraging the network to learn robust and informative temporal features from the raw accelerometer data. The three tasks, each implemented as a classification problem with an additional softmax layer on top of the shared feature extractor $f_{\theta }$, are: (1) The time reversal detection task presents the model with both an original accelerometry sequence and its time-reversed counterpart, requiring it to determine whether the temporal order has been preserved. This forces the network to capture directional patterns in movement data. (2) In the segment permutation recognition task, each input window is divided into *K* non-overlapping segments whose positions are randomly shuffled. The model then predicts the original ordering of the segments, which promotes the learning of temporal dependencies at a coarser scale. (3) The time warping identification task involves applying a monotonic but nonlinear distortion to the time axis of the sequence and challenging the model to distinguish between warped and unwarped samples. This trains the representation to be sensitive to subtle temporal dynamics that may be altered by warping.

The composite SSL objective combines these tasks with balanced weights:

$$\begin{aligned} \mathcal {L}_{\text {SSL}} = \lambda _1\mathcal {L}_\text {rev} + \lambda _2\mathcal {L}_\text {perm} + \lambda _3\mathcal {L}_\text {warp}, \end{aligned}$$

where $\mathcal {L}_{\text {rev}},\ \mathcal {L}_{\text {perm}},\ \mathcal {L}_{\text {warp}}$ denote the loss for time-reversal detection, segment permutation recognition, and time-warping identification, respectively, and $\lambda _{1},\ \lambda _{2},\ \lambda _{3}$ are nonnegative weights as hyperparameters.

After the SSL, the second step of self-supervised learning framework for sleep stage classification is the downstream sleep stage classification using labeled data, where the pre-trained feature extractor $f_\theta$ was then fine-tuned with a deep recurrent network $c_\omega$ (SleepNet, including a ResNet-17 V2 (He et al. 2016) with 1D convolution for feature extraction, a bi-directional Long-Short-Term-Memory (LSTM) network for temporal dependencies learning (Huang et al. 2015) and two fully-connected layers for sleep stage prediction) to train a sleep-stage classifier $c_\omega$ on polysomnography-labeled accelerometer data, minimizing the cross-entropy loss:

$$\begin{aligned} \mathcal {L}_\text {stage} = -\mathbb {E}_{(X,y)}\left[ \sum _{s=1}^5 y_s \log \sigma (c_\omega (f_\theta (X))_s\right] , \end{aligned}$$

where $y = (y_1, \ldots , y_5)$ is the one-hot encoded ground-truth sleep stage label for five different stages of sleep, including wake, REM, and three stages of non-REM, $\sigma (\cdot )$ is the softmax function, and $c_\omega (f_\theta (X))_s$ denotes the output logit for sleep stage *s*.

This approach for classifying sleep stages leverages the generalization benefits of self-supervised learning using massive unlabeled accelerometer data from UK Biobank while adapting to the sleep-stage labeling task using limited polysomnography including 1113 subjects from the Raine Study and the Newcastle cohort, where the sleep labels were aligned with its concurrent accelerometer data as the model ground truth. Internal validation was performed yielding F1 scores of around 0.75 using a pooled two-class setting (sleep/wake) and 0.57 in the pooled three-class setting (wake/REM/non-REM), respectively. External validation was performed on 53 polysomnography nights from the Leicester and Pennsylvania cohorts, with F1 scores 0.66 and 0.49 in the two settings. These validated sleep stage predictions, combined with physical activity labels that predominantly characterize nonsleep periods, form the complete set of labeled temporal sequences used for our downstream modeling of behavioral dynamics for identifying signals indicative of prodromal PD.

## C. Additional exploratory plots

For clarity of presentation, only one prodromal group is shown in the main text; plots for the remaining groups are provided here. While the diagnosed group generally exhibits lower acceleration than the randomly subsampled censored group, the differences become less distinct, and sometimes inconsistent, when comparing the censored group with the prodromal subgroups (See Figure 7, 8, 9, 10, 11).
